# Baclofen-Assisted Alcohol Detoxification with Minimal Diazepam Dosing: A Controlled Study at Corfu General Hospital Detoxification Unit

**DOI:** 10.1192/j.eurpsy.2025.431

**Published:** 2025-08-26

**Authors:** M. Demetriou, P. Argitis, V. Anagnostopoulou, M. Peyioti, F. Mourka, V. Markatis, Z. Chaviaras

**Affiliations:** 1 Psychiatry Department, General Hospital of Corfu, Corfu; 2 University General Hospital of Alexandroupolis, Alexandroupolis, Greece

## Abstract

**Introduction:**

Alcohol withdrawal syndrome is a significant challenge in the management of alcohol use disorder, with traditional treatments often relying heavily on benzodiazepines like diazepam. This study aimed to explore the efficacy and safety of incorporating baclofen (30 mg/day) alongside minimal, tailored diazepam doses—adjusted according to alcohol intake and CIWA-Ar scores—to manage withdrawal symptoms more effectively than conventional diazepam protocols. By reducing the total diazepam needed and shortening detoxification time, the study highlights the potential of baclofen to offer a faster, safer approach to alcohol withdrawal treatment.

**Objectives:**

To evaluate the efficacy and safety of combining baclofen (30 mg/day) with minimal diazepam doses—calculated based on alcohol consumption and adjusted by CIWA-Ar scores—in managing alcohol withdrawal symptoms more rapidly than standard diazepam protocols.

**Methods:**

Sixty-nine patients with alcohol use disorder were enrolled and randomized into two groups. The baclofen group (n = 32) received baclofen 30 mg/day plus minimal diazepam, with initial diazepam doses based on average daily alcohol units consumed (1 mg diazepam per unit) and adjusted using CIWA-Ar scores. The standard group (n = 37) received a conventional diazepam-based detoxification regimen with fixed starting doses adjusted by withdrawal symptoms. Primary outcomes were the total diazepam dosage required and the duration of detoxification. Secondary outcomes included daily CIWA-Ar scores and incidence of adverse effects. Statistical analyses employed independent t-tests and chi-square tests, with p < 0.05 considered significant.

**Results:**

The baclofen group required significantly less diazepam compared to the standard group (mean total dose: 30 ± 10 mg vs. 50 ± 15 mg; p < 0.001). They also experienced a shorter detoxification duration (mean: 15 ± 1 days vs. 19 ± 1 days; p = 0.01), indicating a faster withdrawal process. CIWA-Ar scores were consistently lower in the baclofen group throughout detoxification (mean: 6 ± 2 vs. 10 ± 3; p < 0.001), reflecting milder withdrawal symptoms. No significant adverse effects were observed in either group, including over-sedation, respiratory depression, or hallucinations.

**Conclusions:**

Combining baclofen (30 mg/day) with minimal diazepam—calculated based on alcohol consumption and adjusted by CIWA-Ar scores—effectively controlled alcohol withdrawal, reduced diazepam use by 40%, and shortened detoxification by about four days. The protocol was well-tolerated and may benefit patients at risk from high benzodiazepine doses or in settings aiming to limit benzodiazepine use. These findings suggest baclofen can reduce medication needs and speed up recovery. Larger trials are needed to confirm these results and evaluate long-term outcomes like relapse rates and sustained abstinence.

**Disclosure of Interest:**

None Declared

